# Características Clínicas da Hipertensão Arterial Resistente vs. Refratária em uma População de Hipertensos Afrodescendentes

**DOI:** 10.36660/abc.20190218

**Published:** 2020-07-28

**Authors:** Cristiano Macedo, Roque Aras, Isabella Sales de Macedo

**Affiliations:** 1 Hospital Universitário Professor Edgard Santos SalvadorBA Brasil Hospital Universitário Professor Edgard Santos – Cardiologia,Salvador- BA - Brasil; 2 Universidade de São Paulo Faculdade de Medicina São PauloSP Brasil Universidade de São Paulo - Faculdade de Medicina,São Paulo, SP – Brasil

**Keywords:** Hipertensão/complicações, Grupo com Ancestrais do Continente Africano/genética, Estudo Comparativo, Epidemiologia, Infarto do Miocárdio, Acidente Vascular Cerebral

## Abstract

**Fundamentos:**

Afrodescendentes têm sido associados a uma maior gravidade da hipertensão arterial e maior incidência de complicações cardiovasculares. Características na apresentação da hipertensão resistente (HR) ou hipertensão refratária (HRf), especificamente nessa etnia, não têm sido devidamente estudadas.

**Objetivos:**

O estudo compara características clínicas e epidemiológicas e prevalência de eventos cardiovasculares em afrodescendentes com diagnóstico de HR ou de HRf. Métodos: Estudo transversal realizado em ambulatório de referência para pacientes com Hipertensão Grave. O nível de significância foi de 5%.

**Resultados:**

Avaliados 146 pacientes consecutivos, dos quais 68,7% eram do sexo feminino. A média de idade foi de 61,8 anos, sendo 88,4% afrodescendentes (pardos ou negros). 51% apresentavam HRf. Houve alta prevalência de fatores de risco cardiovascular: 34,2% tinham diabetes, 69,4% dislipidemia, 36,1% obesidade e 38,3% história de tabagismo. Função renal reduzida foi observada em 34,2%. Eventos cardiovasculares prévios ocorreram em 21,8% para infarto do miocárdio e em 19,9% para acidente vascular cerebral. O escore de risco de Framingham foi moderado/alto em 61%. Os pacientes com HRf eram mais jovens (média de idade de 59,38±11,69 anos *versus* 64,10±12,23 anos, p=0,02), tinham mais dislipidemia (83,8 *versus* 66,7%, p=0,021) e acidente vascular cerebral (30,4 *versus* 12,3%, p=0,011) quando comparados aos com HR. O uso de combinação de ACEi/BRA+CCB+Diurético, clortalidona e espironolactona também foi mais frequente em indivíduos com HRf.

**Conclusão:**

Afrodescendentes com HR apresentaram alto risco cardiovascular, alta prevalência de HRf, maior frequência de dislipidemia e de acidente vascular cerebral, compatível com alta incidência de lesão a órgãos-alvo. (Arq Bras Cardiol. 2020; 115(1):31-39)

## Introdução

A alta proporção de indivíduos com hipertensão arterial sistêmica (HAS) que não atingem as metas terapêuticas adequadas tem impacto direto sobre a morbidade, a mortalidade, a incapacidade e os custos com saúde.^1 - 3^ Mesmo com o uso adequado de anti-hipertensivos, um número significativo de pacientes permanece com pressão arterial (PA) elevada, condição caracterizada como Hipertensão Resistente (HR) e definida como a persistência da PA elevada apesar do uso de três drogas anti-hipertensivas de diferentes classes, ou quando o controle da PA ocorre apenas com o uso de quatro ou mais drogas, incluindo-se sempre um diurético tiazídico.^2 - 5^ Um subgrupo de pacientes com HR exibe uma apresentação fenotípica de aparente maior gravidade, na qual a PA não é controlada mesmo com o uso de cinco ou mais medicamentos, situação atualmente definida como Hipertensão Refratária (HRf).^2 , 6 - 8^ O uso de uma associação de Inibidor da Enzima Conversora da Angiotensina (IECA) ou Bloqueador do Receptor de Angiotensina (BRA), Bloqueador dos Canais de Cálcio (BCC) e diurético tiazídico tem sido recomendado como a base da terapia farmacológica da HR.^2 , 3 , 5 , 9^

A estimativa da real prevalência da HR é incerta, dificultada pela presença de fatores que determinam a pseudorresistência, como adesão inadequada à terapêutica e ao efeito do jaleco branco.^9 - 12^ Alguns estudos relatam uma proporção de 11 a 33% de hipertensos resistentes entre aqueles com HAS variando de acordo com as características da população e dos critérios de definição.^9 , 13 , 14^ A prevalência de HRf entre os pacientes com HR é ainda menos conhecida, estimada entre 3 e 31% em alguns estudos.^6^

Diferenças fisiopatológicas nos mecanismos envolvidos na resistência ao tratamento da hipertensão na HR e na HRf têm sido descritas.^14^ Alguns estudos apontam para um aparente pior prognóstico, maior prevalência de lesão a órgãos-alvo e risco aumentado de eventos cardiovasculares em pacientes com HR quando comparados a pacientes com hipertensão não resistente.^6 , 15 , 16^ Em indivíduos negros, a hipertensão tende a manifestar-se de forma mais grave, apresentando maior dificuldade de controle e maior probabilidade de complicações e lesão de órgãos-alvo.^17^ Há, no entanto, uma lacuna na literatura na avaliação da associação entre HR e indivíduos de ascendência africana,^18^ a qual pode ser atribuída a fatores genéticos, ambientais ou mesmo fatores locais.^7 , 19 , 20^

O presente estudo tem como objetivo, portanto, comparar características clínicas e epidemiológicas e prevalência de eventos cardiovasculares em afrodescendentes com diagnóstico de HR ou de HRf. Aprimorar o conhecimento dessas características nessa população específica, incluindo aspectos demográficos, sociais, étnicos, condições de acesso a serviços de saúde e distribuição de medicamentos, poderá contribuir para o planejamento de estratégias visando reduzir o impacto negativo dessa importante condição clínica na saúde desses indivíduos.

## Métodos

Trata-se de um estudo transversal, realizado em ambulatório de referência para Doença Cardiovascular Hipertensiva Grave em um Hospital Universitário da cidade de Salvador, Bahia. A população foi composta por pacientes adultos com diagnóstico de HR acompanhados regularmente no ambulatório entre novembro de 2012 e dezembro de 2015. A amostra foi por conveniência, sendo selecionados consecutivamente durante as visitas de rotina todos os pacientes que concordaram em participar do estudo, assinando um termo de consentimento livre e esclarecido. O estudo foi aprovado pelo Comitê de Ética local, estando em conformidade com a resolução 466/12 da Agência Nacional de Saúde Suplementar (ANS).

Foram considerados como tendo HR os pacientes com PA não controlada (pressão arterial sistólica – PAS≥140mmHg e/ou pressão arterial diastólica – PAD≥90mmHg), apesar do uso de três anti-hipertensivos com ações sinérgicas nas doses máximas recomendadas e toleradas, sendo um deles preferencialmente um diurético tiazídico, ou aqueles com PA controlada, utilizando 4 drogas anti-hipertensivas sinérgicas e em doses adequadas, incluindo também um diurético tiazídico.^2^ Pacientes com PAS≥140mmHg e/ou PAD≥90mmHg em uso de cinco ou mais classes de anti-hipertensivos foram considerados portadores de HRf.^7^

A medida da pressão arterial foi realizada durante a consulta médica de rotina, após cinco minutos de repouso, com as costas apoiadas em posição sentada, pernas não cruzadas e o braço apoiado ao nível do coração. Duas medidas foram realizadas, uma antes e outra após a entrevista, com intervalo mínimo de cinco minutos. A média das duas aferições foi utilizada como valor de referência para a PA do paciente. As aferições foram realizadas com um esfigmomanômetro oscilométrico automático Omron HEM 711 DLX, validado pela *British Hypertension Society* (BHS) e pela *Association for Advancement of Medical Instrumentation* (AAMI).^21 , 22^

Uma equipe treinada coletou, através de entrevista estruturada e revisão dos prontuários, informações sobre dados demográficos e clínicos, avaliação clínico-cardiológica, histórico de eventos cardiovasculares, medicamentos, exames laboratoriais e fatores relacionados ao estilo de vida. O risco cardiovascular (RCV) foi estimado pelo escore de risco de Framingham (ERF). A etnia foi autodeclarada de acordo com os padrões brasileiros de branco, preto ou pardo. A presença de eventos cardiovasculares prévios foi definida por história positiva de acidente vascular cerebral (AVC) ou infarto agudo do miocárdio (IAM) relatado pelo participante ou familiar e/ou quando presente no prontuário. A taxa de filtração glomerular (TFG) foi estimada pela equação de Cockcroft-Gault (GFRe-CG).^23^ Para os indivíduos com sobrepeso ou obesidade, utilizou-se o fator de correção sugerido por Saracino et al.,^24^ (GFRe-CGcorrected) A função renal foi considerada anormal quando TFG<60 ml/min. Para a classificação de sobrepeso e obesidade foi considerado o valor de índice de massa corporal (IMC) maior que 25 e 30kg/m^2^, respectivamente.

Como parte do protocolo de atendimento e seguimento, no mínimo uma Monitoração Ambulatorial da Pressão Arterial (MAPA) é realizada para avaliar a possibilidade do efeito do avental branco como causa de possível pseudorresistência ao tratamento da HAS. Para avaliar a adesão à terapia, utilizou-se o questionário de Morisky (MMAS-8). O nível de adesão foi determinado pela pontuação resultante da soma de todos os acertos: alta adesão (8 pontos), média adesão (6 a <8 pontos) e baixa adesão (<6 pontos).^25 , 26^

### Análise estatística

Para análise estatística, foram utilizados o software Microsoft Office Excel 2010 e o SPSS (versão 20.0). Foi realizada uma análise descritiva univariada das características da população investigada e uma análise bivariada (teste do χ^2^ de Pearson) para estimar a associação entre a variável dependente (HR ou HRf) e a variável independente principal (Presença de Aterosclerose, Hipertrofia Ventricular Esquerda e Eventos Cardiovasculares — IAM ou AVC). As variáveis contínuas estudas (PAS, PAD, tempo de diagnóstico de HAS e tempo de seguimento ambulatorial) apresentaram distribuição normal pelo teste de Kolmogorov-Smirnov e foram comparadas entre os grupos HR e HRf através do teste t de *Student* não pareado. As variáveis categóricas têm suas frequências representadas em percentuais e as variáveis contínuas são apresentadas em suas médias e desvio-padrão. O nível de significância admitido foi de 5%.

## Resultados

Foram avaliados 146 pacientes, dos quais 68,7% eram do sexo feminino e 88,4% eram de ascendência africana (pardos e negros), com média de idade de 61,8±12,1 anos. A média de tempo desde o diagnóstico de hipertensão foi de 21,2±12,5 anos (mediana=18 anos), e os pacientes eram acompanhados no ambulatório há uma média de 11,1±8,5 anos (mediana=10 anos). Houve alta prevalência de fatores de risco para doença cardiovascular: 34,2% dos indivíduos apresentou *diabetes mellitus* , 69,4% dislipidemia, 36,1% obesidade, 38,3% história de tabagismo e 61% risco moderado/alto risco para eventos cardiovasculares pelo ERF. História de IAM prévio foi encontrada em 21,8% dos participantes e acidente vascular cerebral em 19,9%. Anormalidade da função renal (TFG<60mL/min) foi identificada em 34,2%. A PAS foi considerada controlada em 29,5% e a PAD em 50,4% da população total estudada, com média de 152,1±28,0 e 88,0±7,6 mmHg, respectivamente, para PAS e PAD. Os participantes utilizaram uma média de 4,8±1,1 agentes anti-hipertensivos, sendo que 80,8% deles receberam prescrição da combinação recomendada de inibidores da ECA ou BRA + BCC + diurético tiazídico, independentemente da associação com outras drogas. Adesão boa ou moderada à terapia de acordo com o questionário MMAS-8 foi encontrada em 61% dos pacientes.

Após a avaliação dos participantes de acordo com a forma fenotípica de apresentação da HAS, 51% foram categorizados como HRf. A idade foi significativamente menor entre os pacientes com HRf quando comparados aos pacientes com HR (média de idade=59,4±11,7 anos *versus* 64,1±12,2 anos, respectivamente, p=0,02). A Tabela 1 mostra a distribuição dos pacientes de acordo com a classificação como HR ou HRf. Em nossa população, o grupo HRf apresentou maior proporção de indivíduos com idade até 60 anos, dislipidemia e história de AVC. Além disso, o grupo HRf apresentou médias mais altas de PA, e o tempo médio de diagnóstico de HAS tendeu a ser maior em pacientes com HRf; no entanto, isso não alcançou significância estatística ( Tabela 2 ).

**Tabela 1:** – Características sociodemográficas e clínicas dos pacientes atendidos em um centro de referência, de acordo com a hipertensão resistente ou refratária

Características			Hipertensão		
			HR	HRf	
	N	%	%	%	Valor de p
**Idade (anos)**					
Até 60	69	46,9	38,0	59,2	
60 ou mais	75	51,0	62,0	40,8	0,012^*^
**Sexo**					
Masculino	46	31,3	32,9	31,0	
Feminino	100	68,7	67,1	69,0	0,724
**Etnia**					
Brancos	12	8,2	12,9	4,3	
Negros	60	40,8	41,4	41,4	
Pardos	70	47,6	45,7	54,3	0,17^†^
**IMC (kg/m** ^**2**^ **)**					
Não obeso	86	58,5	62,3	61,2	
Obeso	53	36,1	37,7	38,8	0,898^‡^
**Diabetes Mellitus**					
Não	86	58,9	56,9	69,0	
Sim	50	34,2	43,1	31,0	0,144
**Tabagismo**					
Não	87	59,6	58,2	63,9	
Sim	54	37,0	41,8	36,1	0,869
**Dislipidemia**					
Não	34	23,1	33,3	16,2	
Sim	102	69,4	66,7	83,8	0,021
**TFG<60mL/min**					
Não	79	54,1	65,5	65,6	
Sim	41	34,2	34,5	34,4	0,275
**Escore de Risco de Framingham**					
Baixo risco	45	30,8	31,3	33,8	
Moderado/Alto risco	89	61,0	68,7	66,2	0,753^§^
**Adesão à terapêutica (MMAS 8)**					
Baixa	44	30,1	33,8	32,4	
Moderada/Alta	89	61.0	66,2	67,6	0,855^//^
**IAM prévio**					
Não	104	71,2	78,5	77,9	
Sim	29	19,9	21,5	22,1	0,942
**AVC prévio**					
Não	105	71,9	87,7	12,3	
Sim	29	19,9	69,6	30,4	0,011
**Uso da tríade (BRA/IECA+BCC+Diurético)**					
Não	30	20,4	25,4	9,9	
Sim	116	79,6	74,6	90,1	0,015

HR: hipertensão resistente; HRf: hipertensão refratária; IMC: índice de massa corporal; TFG: taxa de filtração glomerular; MMAS 8: questionário de Morisky; IAM: infarto agudo do miocárdio; AVC: acidente vascular cerebral; BRA: bloqueador do receptor de angiotensina; IECA: inibidor da enzima conversora de angiotensina; BCC: bloqueador do canal de cálcio. ^*^Valor de P para distribuição de 60 anos ou mais ou menores de 60 anos entre HR versus HRf; ^†^Valor de p para distribuição da etnia entre HR versus HRf; ^‡^Valor de P para distribuição de obesos (IMC≥30) e não obesos (IMC<30) entre HR versus HRf; ^§^Valor de p para distribuição de baixo risco e moderado/alto risco pelo Escore de Risco de Framingham entre HR versus HRf; ^//^Valor de p para distribuição de baixa adesão e moderada/alta adesão pelo escore de Morisky entre HR versus HRf.

**Tabela 2:** – Pressão arterial sistólica, pressão arterial diastólica, tempo de diagnóstico e tempo de seguimento dos pacientes atendidos em um centro de referência, de acordo com a hipertensão resistente ou refratária

Características	Hipertensão		
	HR	HRf	Valor de p
	Média (DP)	Média (DP)	
**PAS (mmHg)**	145,8 (24,8)	158,7 (29,6)	0,008
**PAD (mmHg)**	84,3 (14,1)	92,0 (20,0)	0,012
**Tempo desde o diagnóstico de HAS (anos)**	19,2 (11,9)	23,12 (13,0)	0,078
**Tempo de acompanhamento (anos)**	10,7 (6,1)	11,38 (10,3)	0,665

*PAS: pressão arterial sistólica; PAD: pressão arterial diastólica; HR: hipertensão resistente; HRf: hipertensão refratária; HAS: hipertensão arterial sistêmica; DP: desvio padrão.*

**Figura 1:**
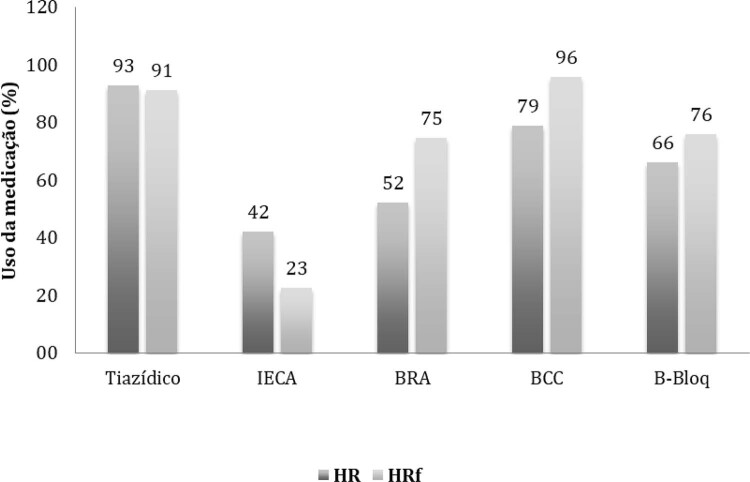
– *Proporção do uso de medicação anti-hipertensiva, de acordo com hipertensão resistente ou refratária. IECA: inibidor da enzima conversora de angiotensina; BRA: bloqueador do receptor de angiotensina; BCC: bloqueador do canal de cálcio; B-Bloq: betabloqueador; HR: hipertensão resistente; HRf: hipertensão refratária.*

**Figura 2:**
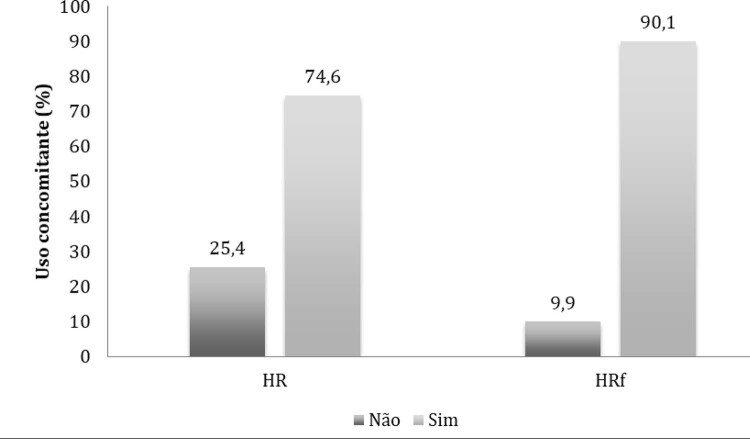
– *Proporção de uso concomitante de drogas anti-hipertensivas (tríade Inibidor da Conversão da Angiotensina ou Bloqueador do Receptor da Angiotensina + Bloqueador do Canal de Cálcio + Diurético), de acordo com hipertensão resistente ou refratária. HR: hipertensão resistente; HRf: hipertensão refratária; P=0,015; para diferença de distribuição do uso da tríade de anti-hipertensivos (IECA ou BRA+BCC+Diurético tiazídico) entre HR versus HRf.*

**Figura 3:**
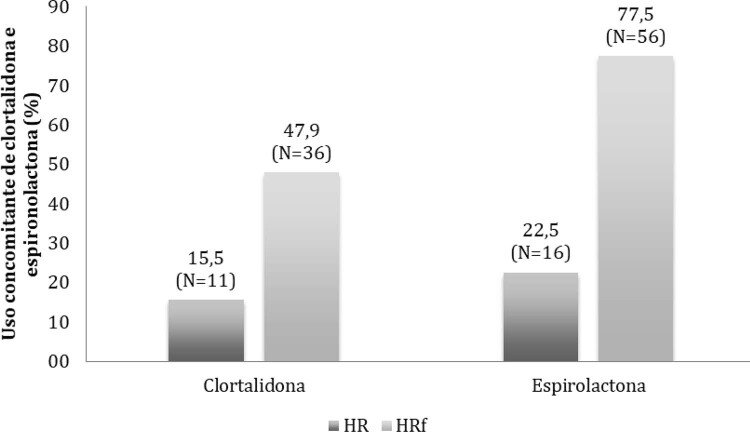
Proporção de uso concomitante de clortalidona e espironolactona, de acordo com hipertensão resistente ou refratária. HR: hipertensão resistente; HRf: hipertensão refratária; P < 0,001; para diferença de frequência do uso de clortalidona entre HR versus HRf; P < 0,001; para diferença de frequência do uso de espironolactona entre HR versus HRf.

Em relação ao uso de anti-hipertensivos, houve maior proporção de uso de BRA e, portanto, menor proporção de uso de IECA. Houve também uma maior frequência de uso de BCC e betabloqueadores em indivíduos com HRf, quando comparados à HR ( Figura 1 ). A Figura 2 mostra que o uso da combinação de ACEi/BRA+BCC+diurético tiazídico foi significativamente maior em pacientes com HRf. A espironolactona foi utilizada por 49,3% dos participantes. Entre os pacientes que utilizaram um diurético tiazídico, 34,5% dos pacientes usaram clortalidona como opção. A Figura 3 mostra que o uso de clortalidona e espironolactona também foi significativamente maior entre indivíduos com HRf.

## Discussão

O grupo de indivíduos predominantemente de ascendência africana com HR é uma população com alto RCV, o que é mostrado por uma alta proporção de participantes (51%) categorizados como HRf, uma apresentação fenotípica associada a uma maior gravidade de HAS de acordo com estudos prévios.^7 , 8 , 16 , 27^ A prevalência de HRf foi estimada em um número limitado de estudos, variando de cerca de 3% em uma população geral de indivíduos com HAS a até 31%, em indivíduos com HR verdadeira com acompanhamento em uma clínica especializada.^7 , 8 , 16^ Esses estudos, no entanto, não usaram uma definição padrão de HRf. Sabe-se que a predominância de etnias negras e pardas está relacionada à gravidade da hipertensão e provavelmente contribuiu para a alta prevalência de HRf em nossa amostra.

A etnia negra tem sido frequentemente associada à HR. Cushman et al.,^28^ relataram uma associação entre etnia afro-americana e resistência ao tratamento anti-hipertensivo, ao avaliar dados do estudo ALLHAT.^28^ Essa associação também foi descrita no estudo brasileiro ELSA, onde a etnia negra foi associada à HR em uma população em tratamento para HAS.^18^ Por sua vez, com base nos dados da coorte do estudo REGARDS, onde a etnia afro-americana foi o principal preditor de HR, Calhoun et al.,^7^ relataram que, em comparação à HR, as razões de prevalência para HRf foram significativamente maiores em negros (RP=3,00; IC 95%=1,68-5,37).^7^ Esses dados apoiam nossos achados de uma alta prevalência de HRf em uma população com a maioria dos indivíduos de ascendência africana. A predominância de pardos e negros em nossa amostra pode ser atribuída ao fato de se tratar de um ambulatório público, atendendo à população de baixa renda, que em nossa região é composta por uma maioria de etnias pardas e negras.

A prevalência de obesidade (36,1%), história de tabagismo (37%), *diabetes mellitus* (34,2%) e dislipidemia (69,4%) reflete uma população de alto RCV, como seria de se esperar em indivíduos com HR. Esse alto RCV em nossa população também é demonstrado pela avaliação do ERF, onde 61% dos indivíduos foram categorizados como risco moderado/alto. Esses achados são consistentes com outros estudos que demonstraram associação de HR com sexo feminino, idade avançada e obesidade^18 , 29^ Calhoun et al.,^7^ relataram um ERF médio de 17,5% em pacientes com HRf e 11,7% em pacientes com HR, com risco de eventos coronarianos e acidente vascular cerebral em 10 anos de 20,8% em HRf e 16,2% em indivíduos com HR, respectivamente.^7^

Também contribuindo para o aumento do RCV na população de nosso estudo, houve uma alta prevalência de função renal anormal, demonstrada por uma TFG estimada de <60ml/min em 34,2% dos indivíduos e uma alta proporção de eventos cardiovasculares prévios (IAM e AVC). Isso sugere que a presença de lesão em órgãos-alvo deve ser frequente nesses pacientes. Muntner et al.,^30^ comparando participantes do estudo ALLHAT com e sem HR, também observaram alto risco de doença coronariana (RR=1,44; IC95%=1,18-1,76), AVC (RR=1,57; IC95%=1,18-2,08) e doença renal em estágio terminal (RR=1,95; IC95%=1,11-3,41) naqueles com HR.^30^

Em nosso estudo, à semelhança de outras publicações,^7 , 8^ houve uma prevalência significativamente maior de AVC prévio em pacientes com HRf, que tiveram PA médias significativamente maiores e uma maior frequência de dislipidemia. A frequência de outros fatores de risco, bem como ERF e adesão terapêutica por MMAS-8 foram semelhantes nos dois subgrupos. Esses dados sugerem que a persistência da PA elevada, provavelmente mais do que os demais fatores de RCV, parece ter um papel fundamental nesse desfecho desfavorável nos portadores de HRf. A hiperatividade simpática, mecanismo proposto para a persistência da PA não controlada em pacientes com HRf,^14^ poderia estar associada a uma maior incidência de AVC nesses indivíduos. A dislipidemia e sua associação íntima com a aterosclerose também podem contribuir negativamente para o prognóstico de pacientes com HRf e precisam ser melhor avaliadas em outros estudos.

Apesar da alta média de idade, observou-se uma proporção significativamente menor de indivíduos com mais de 60 anos entre os pacientes com HRf, apesar da tendência de maior tempo de diagnóstico de HAS dentre estes. Achados semelhantes foram descritos em outros estudos.^14 , 15 , 27^ Esses dados provavelmente estão associados aos possíveis mecanismos imbricados na fisiopatologia da HRf, atribuindo a esse grupo de indivíduos características que implicam no desenvolvimento mais precoce e maior gravidade da HAS.

Em relação ao predomínio do sexo feminino, baseado no estudo ALLHAT, Cushman et al.^28^ encontraram maior dificuldade no controle da HAS em mulheres negras.^28^ Em um estudo populacional realizado na Suécia, Holmqvist et al.,^31^ também relataram que as mulheres tinham uma maior prevalência de HR, exceto quando avaliaram especificamente o subgrupo com HR controlada.^31^ O presente achado de maior proporção de mulheres em nossa amostra de indivíduos com HR deve ser interpretado com cautela, pois pode ser superestimada pelos aspectos culturais brasileiros, uma vez que as mulheres tendem a buscar mais os cuidados com a saúde.^18^ No entanto, esse fato pode identificar um problema que mereça maior atenção, a fim de incentivar uma melhor avaliação clínica e da terapia anti-hipertensiva utilizada nestes indivíduos. Em contraste, alguns autores relataram uma maior prevalência de HR entre os homens.^32^

Em relação à terapia, observou-se um grande uso das várias classes de anti-hipertensivos disponíveis e uma grande proporção de participantes utilizou a combinação de IECA ou BRA, BCC e diurético tiazídico, conforme recomendado na literatura.^2 , 9^ Essa combinação foi prescrita com maior frequência em pacientes com HRf, provavelmente devido à maior dificuldade em obter o controle da PA nesses pacientes. As prescrições de espironolactona como o quarto fármaco a ser introduzido na terapia anti-hipertensiva bem como a opção da clortalidona como o diurético tiazídico de escolha, devido à sua ação mais prolongada, também têm sido recomendadas na literatura.^2 , 3 , 33 , 34^ Alguns autores até sugerem que o uso dessas drogas deva participar dos critérios de definição para HRf.^27^ Em nosso estudo, aproximadamente um terço dos pacientes estava recebendo clortalidona, enquanto quase metade usavam espironolactona. Ambos foram usados significativamente com maior frequência em pacientes com HRf (47,9% dos pacientes com HRf usavam clortalidona e 77,5% espironolactona, versus 11,5 e 22,5% dos HR, respectivamente), o que pode corroborar uma provável classificação adequada de HRf em uma boa parte dos pacientes. A relativamente baixa preferência pela clortalidona como diurético tiazídico pode ser justificada pelo fato de ser um serviço público e a droga não participar da lista governamental de distribuição gratuita de anti-hipertensivos, enquanto a hidroclorotiazida é distribuída gratuitamente. Também é possível que alguns dos participantes não estejam em uso de espironolactona devido a efeitos adversos e/ou contraindicações para esse medicamento. Entretanto, a frequência de uso de clortalidona e espironolactona em nosso trabalho foi, de certa forma, semelhante à de outros estudos.^7 , 8 , 14 , 16^ No entanto, há uma clara necessidade de estimular o uso mais frequente desses medicamentos, que, de acordo com as evidências atuais, seriam mais adequados para o tratamento da HR.

Por sua característica transversal, nosso estudo tem algumas limitações, uma vez que não é possível estabelecer uma relação de causalidade e temporalidade entre algumas associações encontradas, por exemplo, uma maior prevalência de AVC entre aqueles com HRf. Os dados aqui apresentados, no entanto, têm valor em levantar hipóteses a serem comprovadas em estudos longitudinais com maior poder estatístico. Essa amostra de conveniência é derivada de uma população atendida em um ambulatório de referência para pacientes hipertensos graves, com alto RCV, e pode ter superestimado as prevalências e associações descritas. Outro aspecto importante refere-se ao fato de que alguns pacientes com pseudorresistência podem ter sido incluídos, o que também poderia superestimar as prevalências encontradas. No entanto, esses pacientes são acompanhados em um ambulatório específico, a maioria por um longo período (média acima de 10 anos) e com tempo médio de diagnóstico de hipertensão por mais de 20 anos. Eles passam por reavaliações frequentes, incluindo a MAPA^35^ e o escore de Morisky,^25^ para avaliar o efeito do avental branco e a adesão à terapia, respectivamente. Isso poderia minimizar a ocorrência de indivíduos com pseudorresistência nessa amostra. Alguns outros importantes estudos, contudo, têm avaliado pacientes com resistência ao tratamento da HAS, definindo-os como hipertensos resistentes ou refratários aparentes e encontrado associações relevantes.^10 , 36 , 37^ A classificação dos indivíduos quanto à cor da pele foi autorreferida, conforme recomendado em estudos em populações brasileiras que envolvem essa variável,^38^ poderia levar a vieses, devido à grande miscigenação étnica da população brasileira. Contudo, o perfil étnico da amostra estudada é condizente com o da população local e com o da que historicamente frequenta a unidade de saúde onde foi realizado o estudo. A estratificação dos indivíduos como resistentes ou refratários levou em consideração o controle da PA e o número de medicamentos prescritos, desconsiderando se estavam ou não utilizando clortalidona e espironolactona, o que pode, segundo alguns autores, ter superestimado a prevalência de HRf.

## Conclusões

Indivíduos com HR acompanhados nesse ambulatório de referência para tratamento de casos graves de HAS eram em sua maioria afrodescendentes, com alta prevalência de fatores de risco para doenças cardiovasculares e, consequentemente, alto risco cardiovascular segundo o ERF. Encontramos uma alta proporção de indivíduos com a forma mais grave de apresentação fenotípica de resistência ao tratamento da hipertensão, definida como HRf, que apresentou maior frequência de dislipidemia e história de AVC compatível com uma frequência possivelmente maior de dano a órgãos-alvo. Outros estudos mais abrangentes devem ser realizados para melhorar o conhecimento sobre as características dessa população de alto risco, contribuindo para a definição de estratégias adequadas de prevenção e tratamento.
