# Supplementary material for: Características Clínicas da Hipertensão Arterial Resistente vs. Refratária em uma População de Hipertensos Afrodescendentes
Source: Arq Bras Cardiol. 2020 Jul 28;115(1):31–9. [Article in Portuguese] doi: 10.36660/abc.20190218 (PMC8384326; doi:10.36660/abc.20190218)
Supplement: Translation

# Translation: Clinical Characteristics of Resistant vs. Refractory Hypertension in a Population of Hypertensive Afrodescendants

**Authors:** Cristiano Macedo, Roque Aras, Isabella Sales de Macedo

**Affiliations:** 1 Brazil Hospital Universitário Professor Edgard Santos – Cardiologia, Salvador- BA - Brazil; 2 Brazil Universidade de São Paulo - Faculdade de Medicina, São Paulo, SP – Brazil

**Keywords:** Hypertension/complications, African Continental Ancestry Grup/genetic, Comparative Stuy, Epidemiology, Myocardial Infarction, Stroke

## Abstract

**Background:**

Afrodescendants have been associated with a greater severity of arterial hypertension and a higher incidence of cardiovascular complications. Characteristics in the presentation of resistant hypertension (RH) or refractory hypertension (RfH), specifically in this ethnic group, have not been properly studied.

**Objectives:**

The study compares clinical and epidemiological characteristics and prevalence of cardiovascular events in people of African descent diagnosed with RH or RfH.

**Methods:**

Cross-sectional study carried out in a referral clinic for patients with severe hypertension. The level of significance was 5%.

**Results:**

146 consecutive patients were evaluated, of which 68.7% were female. The average age was 61.8 years, with 88.4% of Afrodescendants (mixed race or black). 51% had RfH. There was a high prevalence of cardiovascular risk factors: 34.2% of subjects had diabetes, 69.4% dyslipidemia, 36.1% obesity, and 38.3% history of smoking. Reduced renal function was seen in 34.2%. Previous cardiovascular events occurred in 21.8% for myocardial infarction and in 19.9% for stroke. The Framingham’s risk score was moderate/high at 61%. RfH patients were younger (mean age 59.38±11.69 years versus 64.10±12.23 years, p=0.02), had more dyslipidemia (83.8 versus 66.7%, p=0.021), and stroke (30.4 versus 12.3%, p=0.011) when compared to those with RH. The use of a combination of ACEi/ARB+CCB+Diuretic, chlortalidone and spironolactone was also more frequent in individuals with RfH.

**Conclusion:**

Africandescendant people with RH had a high cardiovascular risk, a high prevalence of RfH, a higher frequency of dyslipidemia and stroke, compatible with a high incidence of injury to target organs. (Arq Bras Cardiol.2020; 115(1):31-39)

## Introduction

The high proportion of individuals with systemic arterial hypertension (SAH) who do not reach the appropriate therapeutic goals has a direct impact on morbidity, mortality, disability and health costs.^1 - 3^ Even with the proper use of antihypertensive drugs, a significant number of patients remain with high blood pressure (BP), a condition characterized as Resistant Hypertension (HR) and defined as the persistence of high BP despite the use of three antihypertensive drugs of different classes, or when BP control occurs only with the use of four or more drugs, always including a thiazide diuretic.^2 - 5^ A subgroup of patients with HR exhibits a phenotypic presentation of apparently greater severity, in which BP is not controlled even with the use of five or more drugs, a situation currently defined as Refractory Hypertension (RfH).^2 , 6 - 8^ The use of an association of Angiotensin Converting Enzyme Inhibitor (ACEI) or Angiotensin Receptor Blocker (BRA), Calcium Channel Blocker (BCC) and thiazide diuretic has been recommended as the basis of HR pharmacological therapy.^2 , 3 , 5 , 9^

The estimate of the real prevalence of RH is uncertain, hampered by the presence of factors that determine pseudoresistance, such as inadequate adherence to therapy and the white coat effect.^9 - 12^ Some studies report a proportion of 11 to 33% of resistant hypertensive patients among those with SAH varying according to the characteristics of the population and the definition criteria.^9 , 13 , 14^ The prevalence of RfH among patients with RH is even less known, estimated between 3 and 31% in some studies.^6^

Pathophysiological differences in the mechanisms involved in resistance to the treatment of hypertension in RH and RfH have been described.^14^ Some studies point to an apparent worse prognosis, higher prevalence of injury to target organs and increased risk of cardiovascular events in patients with RH when compared to patients with non-resistant hypertension.^6 , 15 , 16^ In black individuals, hypertension tends to manifest itself more severely, presenting greater difficulty in control and greater probability of complications and damage to target organs.^17^ There is, however, a gap in the literature in the evaluation of the association between RH and individuals of African descent,^18^ which can be attributed to genetic, environmental or even local factors.^7 , 19 , 20^

The present study aims, therefore, to compare clinical and epidemiological characteristics and prevalence of cardiovascular events in people of African descent diagnosed with RH or RfH. Improving the knowledge of these characteristics in this specific population, including demographic, social, ethnic aspects, conditions of access to health services and distribution of medicines, may contribute to the planning of strategies aimed at reducing the negative impact of this important clinical condition on the health of these individuals.

## Methods

This is a cross-sectional study, carried out in a reference outpatient clinic for Severe Hypertensive Cardiovascular Disease at a University Hospital in the city of Salvador, Bahia. The population consisted of adult patients with a diagnosis of RH followed up regularly at the clinic between November 2012 and December 2015. The sample was made by convenience, being consecutively selected during routine visits all patients who agreed to participate in the study, signing an informed consent form. The study was approved by the local Ethics Committee, complying with resolution 466/12 of the National Supplementary Health Agency ( *Agência Nacional de Saúde Suplementar* – ANS).

Patients with uncontrolled BP (systolic blood pressure – SBP 140mmHg and/or diastolic blood pressure – DBP 90mmHg) were considered as having HR, despite the use of three antihypertensive drugs with synergistic actions at the maximum recommended and tolerated doses, being one of them preferably a thiazide diuretic, or those with controlled BP, using 4 synergistic antihypertensive drugs and in adequate doses, including also a thiazide diuretic.^2^ Patients with SBP≥140mmHg and/or DBP≥90mmHg using five or more classes of antihypertensive drugs were considered to have RfH.^7^

Blood pressure was measured during a routine medical consultation, after five minutes of rest, with the back supported in a sitting position, legs not crossed and the arm supported at heart level. Two measurements were taken, one before and one after the interview, with a minimum interval of five minutes. The average of the two measurements was used as a reference value for the patient’s BP. The measurements were performed with an Omron HEM 711 DLX automatic oscillometric sphygmomanometer, validated by the British Hypertension Society (BHS) and the Association for Advancement of Medical Instrumentation (AAMI).^21 , 22^

A trained team collected, through a structured interview and review of medical records, information on demographic and clinical data, clinical-cardiological evaluation, history of cardiovascular events, medications, laboratory tests and factors related to lifestyle. Cardiovascular risk (CVR) was estimated by the Framingham risk score (FRS). Ethnicity was self-declared according to Brazilian standards of white, black or brown. The presence of previous cardiovascular events was defined by a positive history of stroke (stroke) or acute myocardial infarction (AMI) reported by the participant or family member and/or when present in the medical record. The glomerular filtration rate (GFR) was estimated using the Cockcroft-Gault equation (GFRe-CG).^23^ For individuals with overweight or obesity, the correction factor suggested by Saracino et al. (GFRe-CGcorrected).^24^ Renal function was considered abnormal when GFR<60 ml/min. For the classification of overweight and obesity, the value of body mass index (BMI) greater than 25 and 30 kg/m^2^, respectively, was considered.

As part of the care and follow-up protocol, at least an Ambulatory Blood Pressure Monitoring (ABPM) is performed to assess the possibility of the white coat effect as a cause of possible pseudo-resistance to the treatment of SAH. The Morisky questionnaire (MMAS-8) was used to assess adherence to therapy. The level of adherence was determined by the score resulting from the sum of all correct answers: high adherence (8 points), average adherence (6 to <8 points) and low adherence (<6 points).^25 , 26^

### Statistical analysis

For statistical analysis, Microsoft Office Excel 2010 software and SPSS (version 20.0) were used. A univariate descriptive analysis of the characteristics of the investigated population and a bivariate analysis (Pearson’s χ^2^test) were performed to estimate the association between the dependent variable (RH or RfH) and the main independent variable (Presence of Atherosclerosis, Left Ventricular Hypertrophy and Events Cardiovascular – AMI or stroke). The continuous variables studied (SBP, DBP, time of diagnosis of SAH and time of outpatient follow-up) showed normal distribution by the Kolmogorov-Smirnov test and were compared between the RH and RfH groups using the unpaired Student’s *t* -test. Categorical variables have their frequencies represented in percentages and continuous variables are presented in their means and standard deviation. The level of significance was set at 5%.

## Results

146 patients were evaluated, of which 68.7% were female and 88.4% were of African descent (mixed race and black), with a mean age of 61.8 ± 12.1 years. The mean time since the diagnosis of hypertension was 21.2 ± 12.5 years (median = 18 years), and patients had been followed at the clinic for an average of 11.1 ± 8.5 years (median = 10 years) ). There was a high prevalence of risk factors for cardiovascular disease: 34.2% of individuals had *diabetes mellitus* , 69.4% dyslipidemia, 36.1% obesity, 38.3% history of smoking and 61% moderate risk/high risk for events cardiovascular diseases by the FRS. History of previous AMI was found in 21.8% of participants and stroke in 19.9%. Abnormality of renal function (GFR<60mL/min) was identified in 34.2%. SBP was considered controlled in 29.5% and DBP in 50.4% of the total population studied, with an average of 152.1±28.0 and 88.0±7.6 mmHg, respectively, for SBP and DBP. Participants used an average of 4.8±1.1 antihypertensive agents, 80.8% of whom received a prescription for the recommended combination of ACE inhibitors or ARB + CCB + thiazide diuretic, regardless of the association with other drugs. Good or moderate adherence to therapy according to the MMAS-8 questionnaire was found in 61% of patients.

After evaluating the participants according to the phenotypic presentation of SAH, 51% were categorized as RfH. Age was significantly lower among patients with RfH when compared to patients with RH (mean age=59.4±11.7 years *versus* 64.1±12.2 years, respectively, p=0.02). Table 1 shows the distribution of patients according to the classification as RH or RfH. In our population, the RfH group had a higher proportion of individuals aged up to 60 years, dyslipidemia and a history of stroke. In addition, the RfH group had higher mean BP, and the mean time of diagnosis of SAH tended to be longer in patients with RfH; however, this did not reach statistical significance ( Table 2 ).

**Table 1:** – Sociodemographic and clinical characteristics of patients seen at a referral center, according to resistant or refractory hypertension

Characteristics			Hypertension		
			RH	RfH	
	N	%	%	%	p-value
**Age (years)**					
Up to 60	69	46.9	38.0	59.2	
60 or more	75	51.0	62.0	40.8	0.012^*^
**Gender**					
Male	46	31.3	32.9	31.0	
Female	100	68.7	67.1	69.0	0.724
**Ethnicity**					
White	12	8.2	12.9	4.3	
Black	60	40.8	41.4	41.4	
Brown	70	47.6	45.7	54.3	0.17^†^
**BMI (kg/m** ^**2**^ **)**					
Not obese	86	58.5	62.3	61.2	
Obese	53	36.1	37.7	38.8	0.898^‡^
**Diabetes Mellitus**					
No	86	58.9	56.9	69.0	
Yes	50	34.2	43.1	31.0	0.144
**Smoking**					
No	87	59.6	58.2	63.9	
Yes	54	37.0	41.8	36.1	0.869
**Dyslipidemia**					
No	34	23.1	33.3	16.2	
Yes	102	69.4	66.7	83.8	0.021
**GFR<60mL/min**					
No	79	54.1	65.5	65.6	
Yes	41	34.2	34.5	34.4	0.275
**Framingham’s risk score**					
Low risk	45	30.8	31.3	33.8	
Moderate/high risk	89	61.0	68.7	66.2	0.753^§^
**Adherence to therapy (MMAS 8)**					
Low	44	30.1	33.8	32.4	
Moderate/High	89	61.0	66.2	67.6	0.855^//^
**Previous AMI**					
No	104	71.2	78.5	77.9	
Yes	29	19.9	21.5	22.1	0.942
**Previous stroke**					
No	105	71.9	87.7	12.3	
Yes	29	19.9	69.6	30.4	0.011
**Use of the triad (ARB/ACEi+CCB+Diuretic)**					
No	30	20.4	25.4	9.9	
Yes	116	79.6	74.6	90.1	0.015

RH: resistant hypertension; RfH: refractory hypertension; BMI: body mass index; GFR: glomerular filtration rate; MMAS 8: morisky questionnaire; AMI: acute myocardial infarction; ARB: angiotensin receptor blocker; ACEi: angiotensin converting enzyme inhibitor; CCB: Calcium channel blocker.^*^P-value for distribution of subjects aged 60 years old or older or less than 60 years into RH versus RfH;^†^P-value for ethnicity distribution into RH versus RfH;^‡^P-value for the distribution of obese (BMI≥30) and non-obese (BMI<30) subjects into RH versus RfH;^§^P-value for low and moderate/high risk distribution using the Framingham’s Risk Score into RH versus RfH;^//^P-value for low and moderate/high adherence distribution by the Morisky score into RH versus RfH.

**Table 2:** – Systolic blood pressure, diastolic blood pressure, diagnosis time and follow-up time for patients seen at a referral center, according to resistant or refractory hypertension

Characteristics	Hypertension		
	RH	RfH	p- value
	Mean (SD)	Mean (SD)	
**SBP (mmHg)**	145.8 (24.8)	158.7 (29.6)	0.008
**DBP (mmHg)**	84.3 (14.1)	92.0 (20.0)	0.012
**Time since SAH diagnosis (years)**	19.2 (11.9)	23.12 (13.0)	0.078
**Follow-up time (years)**	10.7 (6.1)	11.38 (10.3)	0.665

*SBP: systolic blood pressure; DBP: diastolic blood pressure; RH: resistant hypertension; RfH: refractory hypertension; SAH: systemic arterial hypertension; SD: standard deviation.*

Regarding the use of antihypertensive drugs, there was a higher proportion of use of ARB and, therefore, a lower proportion of use of ACEi. There was also a higher frequency of use of CCB and beta-blockers in individuals with RfH, when compared to RH ( Figure 1 ). Figure 2 shows that the use of the ACEi/BRA+BCC+thiazide diuretic combination was significantly higher in patients with RfH. Spironolactone was used by 49.3% of the participants. Among patients who used a thiazide diuretic, 34.5% of patients used chlortalidone as an option. Figure 3 shows that the use of chlortalidone and spironolactone was also significantly higher among individuals with RfH.

**Figure 1:** – *Proportion of use of antihypertensive medication, according to resistant or refractory hypertension. ACEI: angiotensin-converting enzyme inhibitor; BRA: angiotensin receptor blocker; BCC: calcium channel blocker; B-Block: beta blocker; RH: resistant hypertension; HRf: refractory hypertension.*

**Figure 2:** – *Proportion of concomitant use of antihypertensive drugs (Angiotensin Conversion Inhibitor triad or Angiotensin Receptor Blocker + Calcium Channel Blocker + Diuretic), according to resistant or refractory hypertension. HR: resistant hypertension; HRf: refractory hypertension; P = 0.015; for difference in distribution of the use of the triad of antihypertensive drugs (ACEI or BRA + BCC + Thiazide diuretic) between HR versus HRf.*

**Figure 3:** Proportion of concomitant use of chlortalidone and spironolactone, according to resistant or refractory hypertension. HR: resistant hypertension; HRf: refractory hypertension; P <0.001; for the difference in frequency of the use of chlortalidone between HR versus HRf; P <0.001; for difference in frequency of spironolactone use between HR versus HRf.

## Discussion

The group of individuals predominantly of African descent with RH is a population with high CVR, which is shown by a high proportion of participants (51%) categorized as RfH, a phenotypic presentation associated with greater severity of SAH according to previous studies.^7 , 8 , 16 , 27^ The prevalence of RfH has been estimated in a limited number of studies, ranging from about 3% in a general population of individuals with SAH to up to 31%, in individuals with true RH with follow-up in a specialized clinic.^7 , 8 , 16^ These studies, however, did not use a standard definition of RfH. It is known that the predominance of black and brown ethnicities is related to the severity of hypertension and probably contributed to the high prevalence of RfH in our sample.

Black ethnicity has often been associated with RH. Cushman et al.^28^ reported an association between African American ethnicity and resistance to antihypertensive treatment, when evaluating data from the ALLHAT study.^28^ This association was also described in the Brazilian study ELSA, where black ethnicity was associated with RH in a population undergoing treatment for SAH.^18^ In turn, based on data from the REGARDS study cohort, where African-American ethnicity was the main predictor of RH, Calhoun et al.^7^ reported that, compared to RH, the prevalence ratios for RfH were significantly higher in blacks (PR=3.00; 95% CI=1.68-5.37).^7^ These data support our findings of a high prevalence of RfH in a population with the majority of individuals of African descent. The predominance of browns and blacks in our sample can be attributed to the fact that it is a public outpatient clinic, serving the low-income population, which in our region is composed of a majority of mixed and black ethnicities.

The prevalence of obesity (36.1%), history of smoking (37%), *diabetes mellitus* (34.2%), and dyslipidemia (69.4%) reflects a population with a high CVR, as would be expected in individuals with RH. This high CVR in our population is also demonstrated by the evaluation of the FRS, where 61% of individuals were categorized as at moderate/high risk. These findings are consistent with other studies that demonstrated an association of RH with female gender, advanced age, and obesity.^18 , 29^ Calhoun et al.^7^ reported an average FRS of 17.5% in patients with RfH and 11.7% in patients with RH, with risk of coronary events and stroke in 10 years of 20.8% in RfH and 16.2% in individuals with RH, respectively.^7^

Also contributing to the increase in CVR in the population of our study, there was a high prevalence of abnormal renal function, demonstrated by an estimated GFR of <60ml/min in 34.2% of the individuals and a high proportion of previous cardiovascular events (AMI and stroke). This suggests that the presence of target organ damage should be frequent in these patients. Muntner et al.,^30^ by comparing ALLHAT study participants with and without RH, also observed a high risk of coronary disease (RR=1.44; 95% CI=1.18-1.76), stroke (RR=1.57; 95% CI= 1.18-2.08), and end-stage kidney disease (RR=1.95; 95% CI=1.11-3.41) in those with RH.^30^

In our study, like other publications,^7 , 8^ there was a significantly higher prevalence of previous stroke in patients with RfH, who had significantly higher mean BP and a higher frequency of dyslipidemia. The frequency of other risk factors, as well as FRS and therapeutic adherence by MMAS-8 were similar in the two subgroups. These data suggest that the persistence of high BP, probably more than the other factors of CVR, seems to have a fundamental role in this unfavorable outcome in patients with RfH. Sympathetic hyperactivity, a mechanism proposed for the persistence of uncontrolled BP in patients with RfH,^14^ could be associated with a higher incidence of stroke in these individuals. Dyslipidemia and its intimate association with atherosclerosis can also contribute negatively to the prognosis of patients with HRf and need to be better evaluated in other studies.

Despite the high average age, a significantly lower proportion of individuals over 60 years of age was observed among patients with RfH, despite the trend for longer time of SAH diagnosis among them. Similar findings have been described in other studies.^14 , 15 , 27^ These data are probably associated with the possible mechanisms involved in the pathophysiology of RfH, attributing to this group of individuals characteristics that imply in the earlier development and greater severity of SAH.

Regarding the predominance of females, based on the ALLHAT study, Cushman et al.^28^ found greater difficulty in controlling SAH in black women.^28^ In a population study carried out in Sweden, Holmqvist et al.^31^ also reported that women had a higher prevalence of RH, except when they specifically assessed the subgroup with controlled RH.^31^ The present finding of a higher proportion of women in our sample of individuals with HR should be interpreted with caution, as it can be overestimated by Brazilian cultural aspects, since women tend to seek more health care.^18^ However, this fact can identify a problem that deserves more attention, in order to encourage a better clinical evaluation and the antihypertensive therapy used in these individuals. In contrast, some authors reported a higher prevalence of RH among men.^32^

Regarding therapy, there was a wide use of the various classes of antihypertensive drugs available and a large proportion of participants used the combination of ACEi or ARB, CCB and thiazide diuretic, as recommended in the literature.^2 , 9^ This combination was prescribed more frequently in patients with RfH, probably due to the greater difficulty in obtaining BP control in these patients. The prescriptions of spironolactone as the fourth drug to be introduced in antihypertensive therapy as well as the option of chlortalidone as the thiazide diuretic of choice, due to its longer duration, have also been recommended in the literature.^2 , 3 , 33 , 34^ Some authors even suggest that the use of these drugs should participate in the definition criteria for RfH.^27^ In our study, approximately one third of patients were receiving chlortalidone, while almost half were using spironolactone. Both were used significantly more frequently in patients with RfH (47.9% of patients with RfH used chlortalidone and 77.5% spironolactone, *versus* 11.5 and 22.5% of those with RH, respectively), which may corroborate a probable adequate RfH classification in a good number of patients. The relatively low preference for chlortalidone as a thiazide diuretic can be justified by the fact that it is a public service and the drug does not participate in the government list of free distribution of antihypertensive drugs, while hydrochlorothiazide is distributed free of charge. It is also possible that some of the participants are not using spironolactone due to adverse effects and/or contraindications for this medication. However, the frequency of use of chlortalidone and spironolactone in our work was somewhat similar to that of other studies.^7 , 8 , 14 , 16^ However, there is a clear need to encourage more frequent use of these drugs, which, according to current evidence, would be more suitable for the treatment of RH.

Due to its transversal characteristic, our study has some limitations, since it is not possible to establish a causality and temporality relationship between some associations found, for example, a higher prevalence of stroke among those with RfH. The data presented here, however, have value in raising hypotheses to be proven in longitudinal studies with greater statistical power. This convenience sample is derived from a population seen at a referral clinic for severe hypertensive patients, with high CVR, and may have overestimated the prevalences and associations described. Another important aspect refers to the fact that some patients with pseudoresistance may have been included, which could also overestimate the prevalences found. However, these patients are followed up in a specific outpatient clinic, most of them for a long period (over 10 years, on average) and with an average time of diagnosis of hypertension for more than 20 years. They undergo frequent reassessments, including ABPM^35^ and the Morisky score,^25^ to assess the white coat effect and adherence to therapy, respectively. This could minimize the occurrence of individuals with pseudoresistance in this sample. Some other important studies, however, have evaluated patients with resistance to the treatment of SAH, defining them as resistant or apparent refractory hypertensive individuals and have found relevant associations.^10 , 36 , 37^ The classification of individuals according to skin color was self-reported, as recommended in studies in Brazilian populations that involve this variable,^38^ could lead to bias, due to the great ethnic mix of the Brazilian population. However, the ethnic profile of the sample studied is consistent with that of the local population and that of those who historically attend the health unit where the study was conducted. The stratification of individuals as resistant or refractory took into account the control of BP and the number of drugs prescribed, disregarding whether or not they were using chlortalidone and spironolactone, which, according to some authors, may have overestimated the prevalence of RfH.

## Conclusions

Individuals with RH followed up at this referral clinic for the treatment of severe cases of SAH were mostly of African descent, with a high prevalence of risk factors for cardiovascular diseases and, consequently, high cardiovascular risk according to the FRS. We found a high proportion of individuals with the most severe form of phenotypic resistance to the treatment of hypertension, defined as RfH, who had a higher frequency of dyslipidemia and a history of stroke compatible with a possibly higher frequency of damage to target organs. Further more comprehensive studies should be carried out to improve knowledge about the characteristics of this high-risk population, contributing to the definition of appropriate prevention and treatment strategies.
